# Neutrophil Oxidized-Modified Proteins and Neutrophil Extracellular Traps in Patients with Community-Acquired Pneumonia

**DOI:** 10.1155/2020/4897038

**Published:** 2020-09-15

**Authors:** Vilen Molotov-Luchanskiy, Altynbek Nukhuly, Larissa Muravlyova, Ryszhan Bakirova, Aruna Kossybayeva, Dmitry Klyuyev, Ludmila Demidchik, Irina Beinikova

**Affiliations:** ^1^“Karaganda Medical University” Non-commercial Joint-Stock Company, Kazakhstan; ^2^Pavlodar State Pedagogical University, Kazakhstan

## Abstract

**Materials and Methods:**

51 patients with CAP were divided into 2 groups depending on the severity of the pathological process. The first group (I) consisted of 32 patients with moderate severity of pneumonia. The second group (II) consisted of 19 patients with severe pneumonia. The third group (III), the comparison group, consisted of 14 CAP patients with chronic obstructive pulmonary disease (COPD). The control group consisted of 19 volunteers.

**Results:**

Statistically significant increase in the level of carbonyl derivatives (CD) in patients of all study groups relative to the control group was revealed. In the group of patients with moderate severity and severe pneumonia, also in CAP patients with COPD, the level of CD exceeded the control group. There was no statistically significant difference in the level of advanced oxidation protein products (AOPP) and myeloperoxidase (MPO) in blood neutrophils between the studied groups.

**Conclusion:**

Results indicate an oxidative imbalance in neutrophils and contribute to the worsening of the course of the disease.

## 1. Introduction

Throughout the world remains a high mortality rate among adult patients with community-acquired pneumonia (CAP). The highest mortality is registered in the older age group with a steady increase in the number of cases of morbidity among persons of working age [1, 2]. The mechanisms of CAP development are diverse and depend both on the etiology of the pathogen and the pathways of penetration into the lungs [3]. The study of changes in the functional and metabolic status of neutrophils is one of the most urgent areas of research, since their condition largely determines the development and outcome of acute pneumonia [4]. Neutrophils are the most numerous group of cells of white blood, the main task of which is the destruction of pathogenic bacteria. It is known that the activation of neutrophils, generation of reactive oxygen species (ROS), and secretion of granules with antimicrobial proteins play a major role in eliminating pathogenic microorganisms. At the same time, the long-term activity of granulocytes makes a significant contribution to the damage of lung tissue, contributing to an unfavorable outcome of pneumonia [5].

Thus, neutrophils play a dual role: on the one hand, they perform a protective function; on the other, they are potential mediators of tissue damage. A sufficient number of studies have been devoted to the study of indices of oxidative stress in the blood to assess the severity of the pathological process, but the number of studies of indices of oxidative stress of neutrophils in CAP patients with chronic obstructive pulmonary disease (COPD) and patients with CAP depending on the degree of disease severity is limited [6, 7]. In this aspect, the study of neutrophils and their functional and metabolic status is one of the most urgent and promising tasks, since their condition largely determines the development and outcomes of the disease.

The work is aimed at studying the indices of oxidative stress and oxidized-modified proteins of neutrophils in CAP patients, depending on the degree of severity and CAP patients with COPD, also comparing the detection frequency of neutrophil extracellular traps in the progression of pneumonia.

To achieve the purpose of the study, an oxidative modification of the neutrophil proteins was evaluated by the level of carbonyl derivatives (CD) and advanced oxidation protein products (AOPP) as a result of oxidative damage of proteins in the cell; also, the activity of the neutrophil enzyme—myeloperoxidase (MPO)—and the presence of neutrophil extracellular traps were studied. All the studied parameters were determined in CAP patients, depending on the severity of the disease (in the acute stage) and compared with the control group of conditionally healthy donors and the comparison group. As a comparison group, the CAP patients with COPD were used.

## 2. Materials and Methods

The study was conducted from the year 2015 to 2016 on the basis of laboratory of the Department of Biochemistry in common with medical institutions, where the diagnosis and collection of blood were conducted for this study. The study included 51 patients diagnosed with community-acquired pneumonia and 14 patients with secondary home pneumonia on a background of COPD of moderate severity and type 2 respiratory failure. At the time of the study, more than 90% of the patients refrained from smoking for two days or more. Prior to the study, informed consent was obtained from all patients and healthy individuals for participation in the study; also, the study was approved by the Ethics Committee of the Karaganda Medical University.

Depending on the severity of the pathological process, CAP patients were divided into 2 groups. The first group (I) consisted of 32 patients with moderate severity pneumonia and type 2 respiratory failure at the age of 27 to 49 years. The second group (II) consisted of patients with severe pneumonia and type 2–3 respiratory failure—19 people aged 21 to 63 years. The third group (III), the comparison group, consisted of 14 CAP patients with COPD in the age range from 32 to 73 years. The control group consisted of 19 nonsmoking, conditionally healthy volunteers without clinical, laboratory, and functional signs of inflammation of the same age range as the patient's groups.

The criteria for inclusion in the study are as follows: community-acquired pneumonia confirmed by the laboratory, instrumental and physical methods, secondary home pneumonia on a background of COPD, age of patients from 18 to 73 years, absence of acute infectious-inflammatory processes of other organs during the study, prolonged (more than 2 weeks) course of pneumonia, and the absence of concomitant pathology of the endocrine system in the stage of decompensation. The exclusion criteria for the study were noninfectious infiltrative lung lesions, such as infarct pneumonia, neoplasms, pulmonary tuberculosis, parasitic invasions, and bronchiectasis.

CAP was diagnosed on the basis of the presence of a pulmonary infiltrate, verified X-ray, and clinical picture (cough with hard-to-separate mucopurulent sputum, chest pain, shortness of breath of mixed character, body temperature up to 38°C). Tachypnea was recorded in the absolute majority of patients. In the CBC, an increase in erythrocyte sedimentation rate, leukocytosis, a shift of the leukocyte formula to the left, and lymphopenia were observed. A bacteriological study of sputum revealed that pneumococci had an etiological role in the development of pneumonia in 79.6% of patients.

After the collection of blood, the upper layer of cells containing leukocytes was collected. The present erythrocytes were destroyed with 0.83% ammonium chloride solution. To wash leukocytes, a phosphate buffer was used. The number of neutrophils was standardized to 1 million in 1 ml of medium. The viability of neutrophils in the test with 0.5% trypan blue was 80%.

The content of carbonyl derivatives (CD) in the neutrophils was determined in the reaction with 2,4-dinitrophenylhydrazine by the method of Levine et al. [8, 9]. The optical density of formed dinitrophenylhydrazones was recorded spectrophotometrically at 370 nm. The AOPP was prepared in vitro as described by Witko-Sarsat et al. [9]. 81% of PBS solution was added to neutrophils in a ratio of 1 : 5, then 200 *μ*l of 15% glacial acetic acid. Immediately at the time of measurement, 100 *μ*l of a 1.16 M KI solution was added, and the absorbance was measured at 340 nm. The activity of myeloperoxidase (MPO) of neutrophils was determined by the method described by Dolgushin et al. [10] which is based on the ability of the enzyme to catalyze the oxidation of indigo carmine with hydrogen peroxide (unit of measure—mcd/l). Detection of neutrophil extracellular traps (NETs) was carried out according to the method described by Dolgushin [10]. The results were expressed in percentage of the number of neutrophils in the field of view. The staining of blood smears was performed according to the May-Grunwald method.

### 2.1. Statistical Analyses

The comparison of the parameters between study groups, due to nonnormal distributions, was carried out using the Kruskal–Wallis analysis of variances (ANOVA) and median test (the differences were considered reliable at significance level *p* < 0.05) and correlation analysis, using Spearman correlation, to determine the relationship between the activity of MPO and the level of AOPP.

## 3. Results

When studying oxidized-modified proteins in blood neutrophils, a statistically significant increase in the level of CD in patients of all study groups relative to the values of the control group (*p* < 0.05) was revealed (Table 1). According to the received data, there were no statistically significant differences in the content of CD depending on the degree of CAP severity, and no significant differences were found with the comparison group. However, we can note the tendency to increase the level of CD in CAP patients with COPD compared to the other studied groups.

Thus, in the group of patients with moderate severity and severe pneumonia, the level of CD was, on average, exceeded than that of the control group by 73% and 89%, respectively, with a maximum value of 1.02 nmol/106 cells in these groups. At the same time, in CAP patients with COPD, the level of CD was 2.9 times higher relative to the values of the control group. In all groups of patients, there were no values below the control.

In this case, we recorded the presence of two clusters of CD in all groups of patients, both CAP and CAP with COPD. The first cluster is the values of the CD of neutrophils that corresponded to those of the control group. The second cluster is the values of CD of neutrophils that exceeded the values of the control group. The investigated groups of patients differed only in the degree of expression of one or another cluster. This suggests that, despite the burden of the disease, there are patients with a normal level of CD that was also shown by us in earlier studies. Perhaps, this is due to the different functional state of neutrophils.

There was no statistically significant difference in the level of AOPP in blood neutrophils between the studied groups. It can only be noted that for patients with moderate severity and severe pneumonia, there is a tendency for an increase in the AOPP level, while for CAP patients with COPD a tendency for decrease relative to the control group (Table 1).

**Figure 1:**
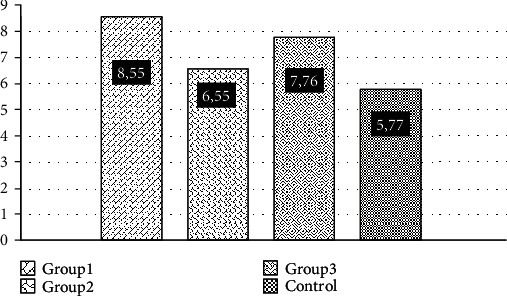
Myeloperoxidase activity in the neutrophils of the patient study groups.

**Figure 2:**
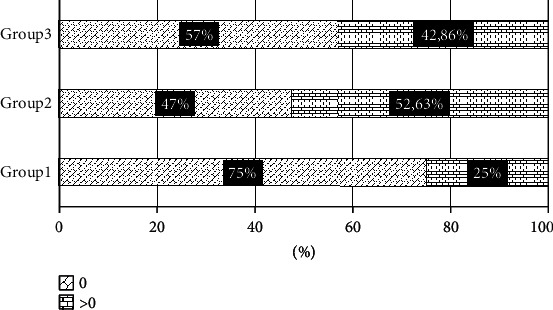
The percentage of patients with different amounts of NET's (per 100 neutrophils).

When studying the activity of neutrophil MPO, according to our data, there was no statistically significant difference between the study groups (Figure 1). In our opinion, this may be due to the fact that the activity of the enzyme depends on the etiology of the disease, since it has been shown that the development of pneumonia from influenza infection reduces MPO activity. Thus, in the work of the authors Nagoev and Betsucova, it was shown that the more severity of influenza-associated pneumonia, the greater inhibition of MPO activity of neutrophils was [11]. According to other data, weighting the course of pneumonia significantly increases the activity of myeloperoxidase [12]. In our study, pneumococci were the dominating etiologic factor in the development of pneumonia, and in 20.4% of the cases, pneumonia was preceded by acute respiratory viral infection and influenza. Therefore, the activity of MPO requires further study depending on the etiology of pneumonia. The percentage ratio of NETs is shown in Figure 2.

The following Figure 2 was revealed in the percentage ratio of NETs among the surveyed groups. In the group of CAP patients with moderate severity, NETs were detected in an amount of 3 to 31 pcs. per 100 neutrophils in 25% of the patients. In group II, NETs were detected in the quantitative range from 1 to 8 pcs. per 100 neutrophils in 52.63% of patients. In the group of patients with pneumonia on the background of COPD, NETs were detected in an amount from 2 to 6 pcs. per 100 neutrophils in 42.86% of the patients. In the control group, neutrophil extracellular traps were absent.

## 4. Discussion

The results we obtained indicate an increase in the activity of oxidative stress and the intensity of formation of oxidized-modified proteins, since CD and AOPP are indicators of the production of free radicals. With the development of inflammation, increased production of ROS by neutrophils in combination with an imbalance of the antioxidant defense system is a condition for the development of oxidative stress [12]. Oxidative stress, in turn, contributes to the damage of cellular structures in inflammatory lung diseases, where the metabolic activity of neutrophils is not limited by the oxygen content [13]. We detected a statistically significant increase in the content of CD and a trend towards an increase in the AOPP level in neutrophils in CAP patients of both moderate and severe severity. The phenomenon of accumulation of modified proteins can be explained by a decrease in the redox potential in cells and a violation of the processes of their proteolytic degradation. In CAP patients with COPD, a tendency towards a decrease in the content of AOPP relative to control with a statistically significant increase in the CD level was detected. The content of AOPP in cells is associated with MPO activity, since it is believed that the hypochlorous acid produced by the enzyme directly participates in the formation of AOPP. In our opinion, in patients with secondary home pneumonia on a background of COPD, the type of oxidative modification of proteins associated with carbonylation predominates, while another type of oxidative modification of proteins, associated with the action of chlorine-containing oxidants and MPO activity, is weakened, which is accompanied by a decrease in AOPP. Neutrophil extracellular traps were found in the blood of patients in the study groups. They were not detected in all patients and their number varied. Thus, with an average severity of pneumonia, the number of traps reached 31 per 100 neutrophils in 25% of patients; in severe pneumonia and pneumonia in COPD, the percentage of patients with NETs increased, but the number of traps did not exceed 8 per 100 neutrophils.

## 5. Conclusion

The appearance of NETs in the blood of patients, in our opinion, is an unfavorable factor, since, in the formation of NETs, extracellular network-like structures containing DNA and histones are formed. It is known that extracellular histones induce cell damage and provoke the formation of thrombin [14, 15]. Thus, the results that we have revealed indicate an oxidative imbalance in neutrophils, which undoubtedly affects the state of the pulmonary parenchyma and contributes to the worsening of the course of the disease.

## Figures and Tables

**Table 1:** The content of oxidized-modified proteins of neutrophils in study groups.

Study groups	CD (nmol/10^6^ cells)			АОРР (*μ*mol/10^6^ cells)		
	Median	Lower quartile	Upper quartile	Median	Lower quartile	Upper quartile
Moderate severity pneumonia (*n* = 32)	0.286^∗^	0.230^∗^	0.429^∗^	0.041	0.031	0.113
Severe pneumonia (*n* = 19)	0.312^∗^	0.251^∗^	0.407^∗^	0.058	0.024	0.108
Secondary home pneumonia on a background of COPD (*n* = 14)	0.494^∗^	0.294^∗^	0.580^∗^	0.029	0.019	0.042
Control (*n* = 19)	0.165	0.147	0.286	0.045	0.032	0.057

CD: carbonyl derivatives; AOPP: advanced oxidation protein products; COPD: chronic obstructive pulmonary disease. ^∗^Reliability of differences with the control, *p* < 0.05.

## Data Availability

The quantitative data used to support the findings of this study are available from the corresponding author upon request.
